# Predictive formula of cervical lordosis in asymptomatic young population

**DOI:** 10.1186/s13018-019-1526-x

**Published:** 2020-01-03

**Authors:** Yuchen Zhu, Zhongcheng An, Yingjian Zhang, Hao Wei, Liqiang Dong

**Affiliations:** 1 Department of Spine Surgery, Second Affiliated Hospital of Zhejiang Chinese Medical University, Hangzhou, 310005 People’s Republic of China; 2 The Second Clinical Medical College of Zhejiang Chinese Medical University, Hangzhou, People’s Republic of China

**Keywords:** Cervical sagittal alignment, Cervical lordosis, Thoracic inlet angle, Regression analysis, Predictive formula

## Abstract

**Background:**

Not a large number of previous studies have reported the normal sagittal balance of the cervical spine and physiological cervical lordosis (CL) has not been clearly defined yet.

**Methods:**

This was a prospective radiological analysis of asymptomatic subjects. The following cervical sagittal parameters were measured: CL, thoracic inlet angle (TIA), T1 slope (T1S), neck tilt (NT), and C2–7 sagittal vertical axis (C2–7 SVA). The *Pearson correlation test* was calculated, and the *stepwise multiple regression analysis* was conducted by using the CL (dependent variable) and the other cervical sagittal parameters (independent variables) to determine the best sets of predictors. A *paired sample t test* was conducted between the predicted and measured values.

**Results:**

The mean age of 307 participants was 24.54 + 3.07. The mean CL, TIA, T1S, NT, and C2–C7 SVA was 17.11° ± 6.31°, 67.87° ± 7.78°, 25.84° ± 5.36°, 42.53° ± 6.68°, and 14.60 ± 8.20 mm, respectively. The formula was established as follows: CL = 0.762 × T1S − 0.392 × C2–C7 SVA + 0.25 × TIA − 13.795 (*R* = 0.812, *R*^2^ = 0.660) (*stepwise multiple regression*) and CL = 0.417 × TIA − 11.193 (*R* = 0.514, *R*^*2*^ = 0.264) (*simple linear regression*). There was no statistical difference between the predicted CL and the measured CL (*t* = 0.034, *P* = 0.973).

**Conclusions:**

There was a significant correlation between CL and other cervical sagittal parameters, including TIA, T1S, NT, and C2–C7 SVA in asymptomatic Chinese population. The results of this study may serve as a normal reference value for the study of asymptomatic population.

## Background

The spine has a certain physiological curvature in the sagittal plane. For the human body always tends to obtain a stable posture at the minimum energy expenditure when standing and walking, it is critical to maintaining the sagittal balance of the spine [1]. The cervical spine, as the most mobile part relative to the rest of the spinal column and also supports the mass of the head, plays a pivotal role in sagittal spinal balance. Any deviations from the normal alignment of the mass of the head would result in a biomechanical imbalance of the cervical spine and an increase in muscular energy expenditure, and bringing a variety of disorders and complications. With the deepening of research, sagittal plane alignment is increasingly recognized as a critical parameter in the setting of adult spinal deformity [2–4]. It has become evident that good clinical outcomes in the treatment of spinal deformity require proper alignment [5]. In recent years, significant progress has been made in the study of the global spinal sagittal alignment parameters. However, the research is largely focused on the spine-pelvic region. Comparatively, the cervical sagittal parameters that affect clinical outcomes of various cervical diseases have not been well defined yet. In addition, there is no set standard to address the amount of correction to be achieved in cervical deformity correction surgery.

However, proper diagnostic evaluation and optimal treatment approaches for spinal deformity need to be based on the investigation of healthy individuals [5]. Herein, we analyzed the cervical sagittal parameters of asymptomatic subjects within a certain age range. The results of this study may serve as a normal reference value of ideal CL for the evaluation of sagittal balance or planning of a fusion angle in the cervical spine.

## Methods

### Study population

From March 2019 to June 2019, the imaging data of volunteers who underwent cervical spine health checkup in our hospital were collected. The study protocol was approved by the Institutional Review Board, and informed consent was obtained from all participants.

The inclusion criteria were as follows: (1) The age was 18–30 years old, (2) no history of symptoms, diagnosis, and treatment related to the whole spine, hip joint, and lower extremity. No history of chronic pain in the neck or shoulder, no history of spinal diseases or surgery, no history of pelvis, hip joint, or lower extremity diseases; (3) no cervical instability and spondylolisthesis showed on X-ray; (4) no significant scoliosis in the coronal plane (Cobb angle < 10°), and no kyphosis deformity in the sagittal plane. A total of 307 volunteers were enrolled, including 144 males and 163 females.

### Radiographic measurement

All the participants had undergone standard anterior and lateral X-rays of the cervical spine. The cervical spine parameters were measured independently using hospital image archiving and communication system (Centricity RIS/PACS, GE healthcare) by two authors. And each result was averaged.

**Fig. 1:**
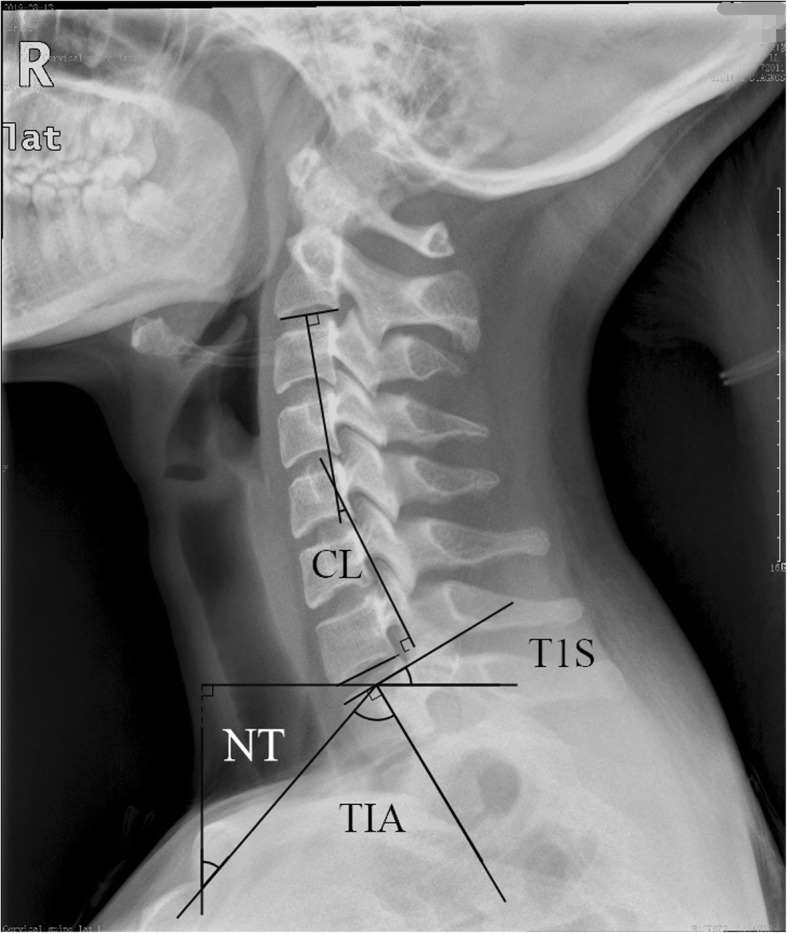
Measurements of the parameters

**Fig. 2:**
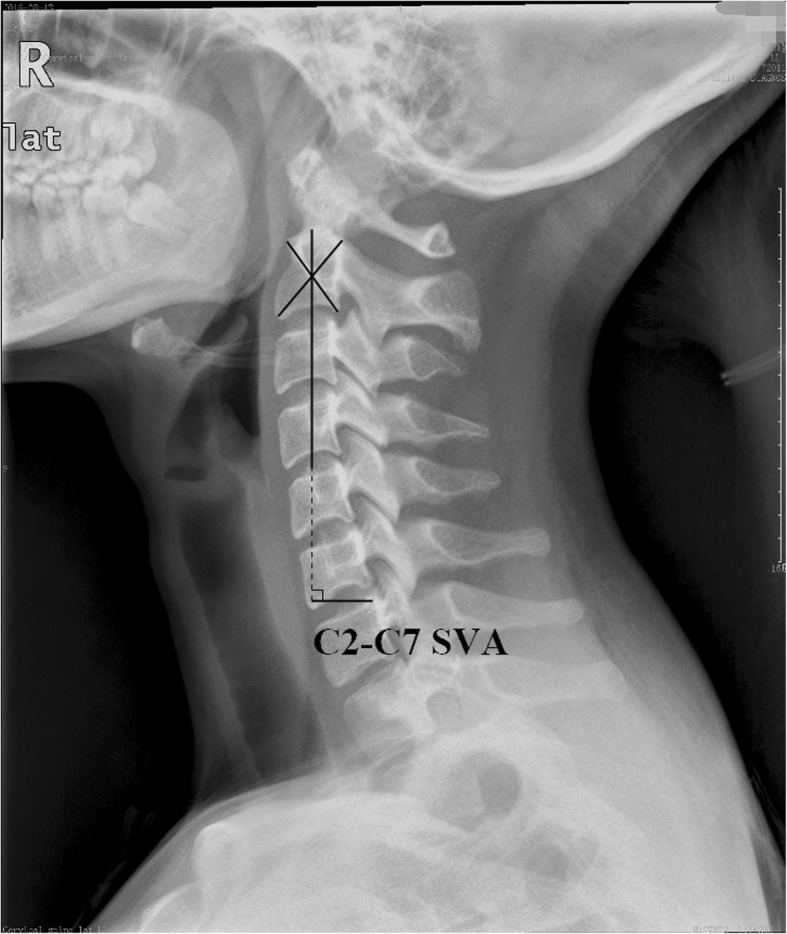
Measurements of the parameters

The parameters are as follows (Figs. 1 and 2, measurements of the parameters).

(1) The thoracic inlet angle (TIA): an angle formed by a line from the center of the T1 upper endplate (T1UEP) vertical to the T1UEP and a line connecting the center of the T1UEP and the upper end of the sternum. (2) The T1 slope (T1S): an angle formed between the horizontal plane and the T1UEP. (3) The neck tilt (NT): an angle formed by a line drawn in the upper end of the sternum and a line connecting the center of the T1UEP and the upper end of the sternum. (4) The cervical lordosis of C2–7 (CL): a Cobb angle between the C2 lower endplate and the C7 lower endplate. (5) The C2–7 sagittal vertical axis (C2–7 SVA): the distance between the C2 plumb line and the posterior C7 upper endplate.

### Statistical analysis

The statistical analyses were performed using SPSS version 20.0 (IBM Corporation, Armonk, NY, USA). There were three steps: (1) descriptive statistics analysis of demographic data and parameters; (2) correlation analysis between CL and other parameters using the Pearson correlation coefficient; (3) stepwise multiple regression and simple regression analysis of parameters, and CL was the dependent variable. A *P* value of < 0.05 was considered statistically significant.

## Results

### Demographic data and sagittal parameters

The mean age of participants was 24.54 + 3.07 (range, 18 to 30 years old). The average value, standard deviation, range, and standard error of CL, TIA, T1S, NT, and C2-C7 SVA were shown in Table 1. The mean CL of 307 participants was 17.11 ± 6.31°, 17.97 ± 6.30° in males and 16.35 ± 6.23° in females. The mean CL in males was larger than that in females (*t* = − 2.246, *P* = 0.025). The mean TIA was 67.87 ± 7.78°, 70.33 ± 9.21° in males, and 65.69 ± 5.40° in females. The TIA in males was significantly higher than that in females (*t* = − 5.457, *P* = 0.000). The results of Pearson correlation analysis between CL and other parameters were shown in Table 2. CL was significantly correlated with T1S, C2–C7 SVA, and TIA (*P* < 0.05), while CL was not correlated with NT (*P* = 0.762).

**Table 1:** Average value, standard deviation, range, and standard error

Parameters	Average value $\Big(\overline{\chi}\pm s$)	Range	Standard error
Age	24.54 ± 3.07	18–30	0.173
CL (degrees)	17.11 ± 6.31	− 8.94–28.44	0.360
TIA (degrees)	67.87 ± 7.78	43.02–88.98	0.444
T1S (degrees)	25.84 ± 5.36	12.00–41.20	0.306
NT (degrees)	42.53 ± 6.68	27.88–59.12	0.381
C2–C7 SVA (mm)	14.60 ± 8.20	− 6.00–40.00	0.468

**Table 2:** Pearson correlation analysis

	CL	*P* value
TIA	0.514	0.000
T1S	0.620	0.000
NT	0.092	0.109
C2–C7 SVA	− 0.196	0.001

### Predictive formula of cervical lordosis

A predictive formula with relevant variables, including TIA, T1S, C2–C7 SVA (NT was excluded during the regression analysis), for CL was established using *stepwise multiple regression analysis* of the above parameters (Tables 3 and 4). That was CL = 0.762 × T1S − 0.392 × C2–C7 SVA + 0.25 × TIA − 13.795 (*R* = 0.812, *R*^*2*^ = 0.660).

**Table 3:** Stepwise multiple regression analysis

Step	*R*	*R*^2^	Adjusted *R*^2^	independent variables
1	0.620	0.384	0.382	T_1_S
2	0.769	0.691	0.588	T_1_S, C_2_–C_7_ SVA
3	0.812	0.660	0.657	T_1_S, C_2_–C_7_ SVA, TIA

**Table 4:** The coefficients and constants of two predictive formulas

Model	Regression coefficients	Standardized coefficients	*P* value
Stepwise multiple regression analysis			
T1S	0.762	0.648	0.000
C_2_–C_7_ SVA	− 0.392	− 0.510	0.000
TIA	0.250	0.308	0.000
Constant	− 13.795	0	0.000
Simple linear regression analysis			
TIA	0.417	0.514	0.000
Constant	− 11.193	0	0.000

In the formula above, T1S and C2-C7 SVA were orientation parameters influenced by the posture. While TIA was a constant morphological parameter, not influenced by the posture and unchanged in adulthood. Thus, a simplified formula for CL was obtained from *simple linear regression analysis* with TIA. That was CL = 0.417 × TIA − 11.193 (*R* = 0.514, *R*^*2*^ = 0.264) (Table 4). Based on the TIA values measured, the predictive value of CL was calculated to be 17.10 ± 3.24°. There was no statistical difference between the predicted value and the measured value using *the paired sample t test* (*t* = 0.034, *P* = 0.973).

## Discussion

The spinal regions (the pelvis and the lumbar, thoracic, and cervical regions) are not independent of one another, and they have multiple significant correlations. As described by Dubousset in his “Conus of Economy” theory, the body adapts to changes in balance in order to regulate the center of gravity over as narrow a perimeter as possible [6]. Sagittal alignment has important implications for muscular energy expenditure in the maintenance of posture. A cervical deformity can lead to compensatory mechanisms such as knee flexion, pelvic retroversion, thoracic hypokyphosis, and lumbar hyperlordosis, to maintain a balanced, upright posture and horizontal gaze [7, 8]. And in cervical spine sagittal parameters, T1S, C2-C7 SVA, chin-brow to vertical angle (CBVA) increases while NT decreases as compensatory mechanisms. Indeed, physiological cervical lordosis is an essential condition for spinal coupling motion. The morphological changes of the cervical sagittal plane will eventually lead to changes in the segment subjected to the greatest stress during the motion and accelerate its degeneration.

The primary purpose of cervical deformity correction surgery is to maintain or restore the horizontal gaze, decompress the spinal cord or nerve root, and reconstruct the cervical spine alignment. There currently exist no commonly acknowledged criteria for cervical sagittal plane correction, but to reconstruct and maintain the global spinal sagittal alignment as far as possible has become a consensus [9, 10]. In recent years, many studies have concentrated on the correlation of cervical alignment parameters to disability scores and myelopathy outcomes. Tang et al. noted that the C1–C2 lordosis angle is an essential parameter for regulating the angle of gaze in cervical reconstructive surgery, and the postoperative C2–C7 sagittal vertical axis (SVA) is significantly correlated with health-related quality-of-life (HRQOL) scores [11]. Ames et al. also suggested correlations between radiographical parameters in the cervical spine and HRQOL outcomes [12]. The authors believed that the TIA and T1S might be used as parameters to evaluate sagittal balance, predict physiological alignment, and guide deformity correction of the cervical spine. According to the formula, geometrically, TIA = T1S + NT, it could be presumed that large TIA increases T1S and finally increase CL to obtain a horizontal gaze and sagittal alignment of the cervical spine with minimum energy expenditure, and vice versa. On the basis of previous studies, we attempt to establish a further relationship between the alignment parameters, that is, a predicting formula.

However, whether spinopelvic parameters or cervical spine parameters, differences exist in population from different regions. The results of the cervical spine parameters of 77 asymptomatic Korean adult volunteers measured by Lee et al. showed that the mean TIA, T1S, and NT were 69.5°, 25.7°, and 43.7°, respectively [13]. Zhang et al. reported that the mean TIA, T1S, and NT of 67 Chinese adults with mild neck symptoms were 72.8°, 22.3°, and 49.7°, respectively [14]. Moreover, radiographic measurements of 120 asymptomatic American adult volunteers performed by Iyer et al. showed the mean TIA, T1S, and NT were 79.8°, 26.1°, and 51°, respectively [15]. Thus, it seems to be significant to investigate correlations of sequential parameters and construct predictive formulas of postoperative spinal alignment of each region in order to plan surgery for cervical deformity correction optimally.

In this study, we observed the cervical sagittal parameters of 307 asymptomatic Chinese adult volunteers, and the mean CL came to 17.11 ± 6.31°. The Pearson correlation coefficient and linear regression models found that CL was significantly correlated with TIA, T1S, NT, and C2-C7 SVA, which was consistent with previous studies [1, 8, 12]. Then, a predictive formula with these relevant variables was made using stepwise multiple regression analysis of the above parameters. The following formula was established, and TIA, T1S, and C2-C7 SVA were important predictive variables: CL = 0.762 × T1S − 0.392 × C2–C7 SVA + 0.25 × TIA − 13.795 (*R* = 0.812, *R*^2^ = 0.660), in which the determination coefficient declared a good linear correlation. However, this formula is based on young asymptomatic adults with normal spinal curvature in the sagittal plane. In pathological conditions, to adapt to the variations in the shape of the spine, several compensation mechanisms are implemented at the segmental, regional, and global levels. Moreover, T1S, as well as C2-C7 SVA, would be influenced, for it is not a constant parameter. Therefore, we consider that it is inefficient to use T1S and C2–C7 SVA to predict the extent of correction to be performed during surgery. In view of the fact that TIA is a constant morphological parameter not influenced by aging or posture, the formula CL = 0.417 × TIA − 11.193 (*R* = 0.514, *R*^2^ = 0.264) could be a simple approach.

Age-related spinal degeneration is one of the crucial factors affecting sagittal spinal parameters [16]. For the purpose of analyzing the relationship between CL and other cervical sagittal parameters in normal individuals, we recruited healthy volunteers of the age-specific group (average age 24.54 ± 3.07) at which most degenerative changes in the spine have not obviously developed. These data can be a useful reference for the study of the asymptomatic population. However, it is also a limitation of our study that we could not see continuous sagittal changes according to all ages because of the certain age range of subjects. Indeed, most of the patients who need surgical intervention are of advanced age. So a further study involving an old age group is valuable for cervical surgery. Another limitation of our study is that we did not take global spinal parameters into consideration. It is recognized that cervical deformity correction should take on a comprehensive approach in assessing global cervical-pelvic relationships [12]. The spinal regions are not independent of one another, and CL as an adaptive spinal segment depends on the alignment of both thoracic and lumbar spine. In previous studies of sagittal spinal parameters, Schwab et al. proposed a simple approach of “lumbar lordosis = pelvic incidence ± 9°”, which has been verified good correlation with HROQL and applied in most reconstructive surgeries at the thoracic and lumbar spine [5, 17, 18]. Therefore, for the cervical spine, further prospective study of the relationship between predictive CL and postoperative HRQOL outcomes is needed to find out whether predictive CL predicted only by cervical sagittal parameters actually can be applied to surgical planning.

## Conclusions

There was a significant correlation between CL and other cervical sagittal parameters including TIA, T1S, NT, and C2-C7 SVA in asymptomatic Chinese population. The results of this study may serve as a normal reference value for the study of asymptomatic population.

## Data Availability

The datasets generated and analyzed during the current study are available from the corresponding author on reasonable request.

## Abbreviations

C2–7 SVA: C2–7 sagittal vertical axis
CBVA: Chin-brow to vertical angle
CL: Cervical lordosis
HRQOL scores: Health-related quality-of-life scores
NT: Neck tilt
T1S: T1 slope
T1UEP: T1 upper endplate
TIA: Thoracic inlet angle
